# Studying the shelf life of butter containing fucoidan, by evaluating sensory and chemical properties

**DOI:** 10.1002/fsn3.3277

**Published:** 2023-03-09

**Authors:** Ahmad Alipour, Mohammad Hossein Marhamatizadeh, Mehdi Mohammadi

**Affiliations:** ^1^ Department of Food Hygiene, Kazerun Branch Islamic Azad University Kazerun Iran; ^2^ Department of Biotechnology, Persian Gulf Studies and Research Center Khalij Fars University Iran

**Keywords:** fucoidan, *Sargassum angustifolium*, sensory properties, shelf‐life, sour cream butter

## Abstract

Fucoidan powder was added in amounts of 0.05, 0.1,0.3, and 0.5% to sour cream butter and sensory and chemical properties were tested on their shelf life for 60 days during storage. Peroxide levels initially increased until day 40 of storage and then decreased. Butter samples from the control group had the highest amount of peroxide on day 40 (15.25 ± 1.41 meq/kg butter), while samples treated with fucoidan 0.5% had the lowest amount of peroxide (6.35 ± 0.53 meq/kg butter). The acidity of butter treatments increased during storage (*p* < .05). Butter samples from the control group had the highest acidity at 60 days of storage (0.40 ± 0.033 mg KOH / g butter), while samples treated with 0.5% fucoidan had the lowest acidity (0.17 ± 0.013 mg KOH / g butter). The treated butter samples showed the highest stability. Fucoidan, as an antioxidant, reduces the taste, odor, and discoloration of butter added with fucoidan during storage because it completely removes odorless tasteless powder, and the free radical chain is involved in oxidation and improves product properties. The results showed that there are no significant changes in the acceptance rate of butter treated with fucoidan during 60 days of storage in the refrigerator (*p* > .05). The sensory scores of the treated butter showed that the sensory properties during the storage period were similar to the control samples, but on day 40 of storage, they decreased. In general, a concentration of 0.5% fucoidan delays the oxidative process and increases shelf life and is selected as a superior treatment in terms of sensory evaluation, and is introduced as a functional food.

## Graphical abstract

Fucoidan powder was added in amounts of 0.05%, 0.1%, 0.03%, and 0.5% to sour cream butter and sensory and chemical properties were tested on their shelf life for 60 days during storage. By increasing the amount of fucoidan in the samples, the amount of acidity and peroxide was affected, and then an acceptable product was produced due to the tastelessness of fucoidan.

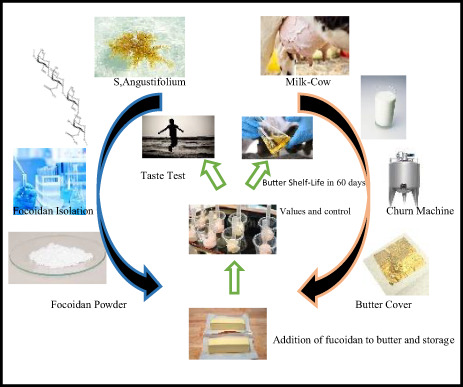

## 1 INTRODUCTION

The most important factor in reducing the shelf‐life of butter is the oxidation process, so the stabilization of butter, which contains large amounts of saturated fatty acids and cholesterol, is of particular importance, so to increase shelf‐life, control of oxidative spoilage is essential (Darmawan et al., 2022). Today, the use of natural antioxidants has increased and the tendency to use them as a substitute for synthetic antioxidants has increased due to their reduced toxicity (Fadda et al., 2022). Antioxidants are widely used as food additives to enhance their properties and protect against oxidative damage in foods and oils (Guan et al., 2021). Algae is a source of biological value and various uses that its use as food and medicine has attracted more attention than any other aspect (Babich et al., 2022). They are one of the richest sources of natural antioxidants and a source of bioactive compounds, and several bioactive compounds, such as antibiotic, antiviral, antifungal, and anticancer effects have been identified from algae, many of which are primary and secondary metabolites. These algae can become active ingredients of interest in the pharmaceutical and food industries (Remya et al., 2022). In a study of 48 species of seaweed, brown algae with the highest antioxidant activity as well as significant amounts of protein with essential amino acid compounds, carbohydrates, vitamins and minerals, and beneficial long‐chain unsaturated fatty acid compounds were measured (El‐Beltagi, Mohamed, et al., 2022; Reyes et al., 2021; Yao et al., 2022). Antioxidant activity is related to molecular weight and sulfate content, which has antibacterial properties and is a strong natural antioxidant (Huang et al., 2018). Fucoidan has probiotic effects and strengthens the immune system. (Huang, Zeng, et al., 2022). So far, a variety of fucoidan compounds have been used in foods, such as beverages, pills, and capsules. Healthy foods can have a positive effect on health beyond basic nutrition (Pradhan et al., 2022). With the therapeutic properties and antioxidant effects extracted from fucoidan, most people agree to use these foods, and considering the therapeutic properties of fucoidan, it is a functional product. This study aims to Study the shelf life of butter containing fucoidan by evaluating the sensory and chemical properties.

## 2 MATERIALS AND METHODS

### 2.1 Study design

All solvents and chemicals used in the present study are made by Merck Company (Merck, Germany) with high analytical purity. The butter samples used in this research were prepared from sour cream and packed in 100 g containers with different grades.

### 2.2 Fucoidan extraction

#### 2.2.1 Sample collection

Samples of *Sargassum angustifolium* were collected from the shores of Bushehr (Persian Gulf, Iran) at a depth of 3–5 m in June to August 2017 and transferred directly to the Marine Biotechnology Laboratory of the Persian Gulf University for fucoidan isolation. Freshly washed seaweed was then air‐dried and turned into a powder.

#### 2.2.2 Extraction of polysaccharides from *S*. *angustifolium*

Fresh *S. angustifolium* was dried in shade and powdered. After that, 20 g of the powdered *S. angustifolium* samples were mixed with 1000 mL ethanol (85%) to remove pigments and proteins and shaken for 12 h at room temperature. Then, the samples were washed with acetone and centrifuged at 49831.6 *g* for 10 min. Then, 5 g of the achieved sample was mixed with 100 mL of distilled water at 65°C for 1 h and centrifuged at 217.828 *g* for 10 min. Then, the supernatant was collected and concentrated using oven. It was then mixed with 1% calcium chloride at 4°C and kept steady overnight for alginate deposition. After centrifuge, the supernatant was collected and mixed with 96% ethanol and stored overnight at 4°C. An achieved polysaccharide materials were then dehydrated by acetone and ethanol (30%) and dried in room temperature. Achieved fucoidan was then filtered (0.45 nm, ALBET‐NY‐045‐47‐BL, Spain) and washed with acetone and ethanol and dried in room temperature (Yang et al., 2008).

#### 2.2.3 Fucoidan isolation

The obtained polysaccharides were then dewatered with acetone and ethanol (30%) and dried at room temperature. The isolated fucoidan was filtered (0.45 nm, ALBET‐NY‐045‐47‐BL, Spain) and washed with acetone and ethanol, and dried at room temperature (Al Monla et al., 2022).

### 2.3 Butter preparation from sour cream

After preparing the yogurt from the fresh milk, the yogurt was kept in the container for a few days to make it sour. Then, the existing cream which was acidified with lactic acid was transferred to the churn machine and after adding water, it was stirred and the resulting butter was collected (Sadri Saeen et al., 2022).

### 2.4 Sample preparation

Fucoidan powder was prepared in concentrations of 0.05%, 0.1%, 0.3%, and 0.5% and added to butter samples. The butter control group did not contain fucoidan content. Samples containing fucoidan and control samples were packed in 100 g containers with two layers of oily toilet paper and then aluminum foil. All samples were then refrigerated. Sampling and sensory testing were performed on days 1, 20, 40, and 60 after storage.

### 2.5 Measurement of oxidative stability

Metrohm Rancimat device was used to determine the induction point of oil mixed with tannic acid. The production of volatile products, such as aldehydes, acids, and alcohols as a result of oil oxidation circulates in the air at a temperature of 110 ± 1.5°C. Then this air is passed through the deionized water and the conductivity of the water is measured. A quantity of 2.5 g of samples was used to measure the thermal resistance of the samples with the Rancimat device (temperature 110–160 degrees Celsius). Samples were analyzed using Rancimat system (version 743) equipped with software, including temperature conditions in the range of 50–220°C and AOCS method 12b‐92 Cd to determine the state of the sphere in terms of its oxidative stability point (Chen et al., 2022).

### 2.6 Sensory properties

The satisfaction of 30 untrained individuals was assessed in several specific periods. The purpose of this study was to evaluate the longevity of butter by emphasizing the appearance, color, taste, and unpleasant odor caused by spoilage. Among the samples, water was used to drink and clean the mouth of the taste of the previous sample (Forde, 2018).

### 2.7 Acidic value

To measure the acidity of butter samples, 20 g of butter was dissolved in 100 mL of the equivalent volume of ethanol chloroform. This solution was titrated in the presence of phenolphthalein until the stable purple color appeared with 0.1 *N* potassium hydroxide (Singh et al., 2022).

### 2.8 Peroxide value

To measure the amount of peroxide, 5 g of butter was dissolved in 30 mL of acetic acid–chloroform (3:2) solution. Then 0.5 mL of saturated potassium iodide solution was added to the previous solution and placed in a dark place for 1 min. After this period, 30 mL of water and 0.5 mL of 1% starch solution were added to it. Then the solution was titrated with 0.01% normal sodium to change the color (Singh et al., 2022).

### 2.9 Statistics

All experiments were carried out in triplicate. Statistical analysis was performed using the SPSS software (Ver 21, USA). Analysis of Variance (ANOVA) and Duncan test were performed to obtain any statistical differences.

## 3 RESULTS AND DISCUSSION

### 3.1 Acid value

Fat exists in the form of triglycerides, and as a result of hydrolysis, acid is produced over a period of time, which is considered an indicator of quality in fat‐rich products. Acidity is a criterion for determining hydrolytic corruption, which increases mainly due to the hydrolysis of fatty acids in the presence of water (Jadhav & Annapure, 2022; Li, Huang, et al., 2022; Nimbkar et al., 2022). However, other oxidation reactions can also lead to the production of free fatty acids. Therefore, acidity monitoring is one of the oxidation quality control parameters. In addition, these compounds can also participate in autoxidation (Ran et al., 2022). Table 1 shows the acidity of the butter samples of the control group and the samples treated with different concentrations of fucoidan during the storage period. In all samples, the acidity increased with time, but the samples containing fucoidan had lower acidity than the control sample, so that the control sample had the highest acidity and the 0.5% sample had the lowest acidity (*p* < .05). The highest amount of acidity on day 60 of the storage period is related to the butter samples of the control group (0.40 ± 0.033 mg KOH/g butter) and the lowest is related to the samples treated with 0.5% fucoidan (0.17 ± 0.013) mg KOH)/gram of butter) (*p* < .05). The use of sources with antioxidant and antimicrobial capacity in natural compounds has increased in recent years due to the decrease in the use of artificial antioxidants, such as butylhydroxyanisole and toluene (El‐Beltagi, Eshak, et al., 2022; López‐García et al., 2022). Natural antioxidants can react with free radicals and suppress them (Akbari et al., 2022). *Sargassum* brown algae has a high potential in terms of antioxidant content and many studies have been conducted on it (Chouh et al., 2022; Polo & Chow, 2022). Fucoidan has antibacterial properties and is a powerful natural antioxidant (Hoang et al., 2022), which, due to this property, prevents the progress of oxidation and the effectiveness of enzymes that catalyze fat hydrolysis (Tatiyaborworntham et al., 2022). The acid value represents the amount of free fatty acids in a food product, the high amount of which causes a decrease in quality (Jadhav et al., 2022; Jamali et al., 2022). The results on the acid value of the samples during the storage time showed that the acid number of the samples increases with the passage of time. The antioxidant obtained from the Cilicica Satureja plant extract was added to the butter and in all the samples, the acidity increased with time, but the samples containing the antioxidant had a lower acidity than the control sample (Adriana & Tiţa, 2020).

**TABLE 1:** Acidic value of butter samples treated with diverse concentrations of fucoidan during the storage period.

Butter treatments	Acidic value (mg KOH/g butter) during the storage period (day)			
	1	20	40	60
Control	0.12 ± 0.011 ^D*a**^	0.19 ± 0.012 ^Ca^	0.28 ± 0.022 ^Ba^	0.40 ± 0.033 ^Aa^
0.05% fucoidan	0.11 ± 0.010 ^Ca^	0.16 ± 0.014 ^Bb^	0.20 ± 0.018 ^Bb^	0.29 ± 0.021 ^Ab^
0.1% fucoidan	0.11 ± 0.010 ^Aa^	0.14 ± 0.012 ^Ab^	0.17 ± 0.016 ^Ac^	0.22 ± 0.020 ^Ac^
0.3% fucoidan	0.10 ± 0.010 ^Aa^	0.13 ± 0.011 ^Ab^	0.15 ± 0.012 ^Ac^	0.18 ± 0.015 ^Ac^
0.5% fucoidan	0.10 ± 0.010 ^Aa^	0.12 ± 0.011 ^Ab^	0.14 ± 0.012 ^Ac^	0.17 ± 0.013 ^Ac^

*Note*: Dissimilar capital letters in each row disclose statistical difference about *p* < .05. Dissimilar small letters in each column disclose statistical difference about *p* < .05.

### 3.2 Peroxide value

Peroxide is the measure of product hydroperoxides that are produced in the early stages of oxidation (Liu et al., 2022). Table 2 shows the amount of peroxide in the control samples and the treated samples with different concentrations of fucoidan during the storage period. The peroxide value of the butter samples of all studied groups increased during the storage period up to day 40 of the storage period (*p* < .05). The highest amount of peroxide on the 40th day of the storage period is related to the butter samples of the control group (20.71 ± 1.65 mEq/kg of butter) and the lowest is related to the samples treated with 0.5% fucoidan (9.10 ± 0.69 mEq per kg of butter) (*p* < .05). The peroxide content of all butter samples decreased from the 40th day of storage to the 60th day. However, the amount of peroxide in the butter samples on the 60th day of the storage period was not lower than on the 20th day of the storage period. Peroxide decreased from day 40 to day 60 after storage. The main reason for this finding could be due to the conversion of primary compounds produced from fat oxidation to secondary components such as malondialdehyde (MDA), which led to a decrease in peroxide levels in butter samples. Peroxide number is an index to measure the amount of fat oxidation and it estimates the peroxides produced by oxidation reactions (Pendyala et al., 2022). Peroxides increase slowly in the initial stages of oxidation, but at the end of the induction period, the concentration of peroxides increases rapidly and then remains constant and then decreases due to decomposition (Fan et al., 2022; Liu et al., 2022).

**TABLE 2:** Peroxide value of butter samples treated with diverse concentrations of fucoidan during the storage period.

Butter treatments	Peroxide value (meq/kg butter) during the storage period (day)			
	1	20	40	60
Control	4.43 ± 0.34 ^D*a**^	11.73 ± 1.01 ^Ca^	20.71 ± 1.65 ^Aa^	15.25 ± 1.41 ^Ba^
0.05% fucoidan	4.10 ± 0.29 ^Da^	9.27 ± 0.85 ^Cb^	16.16 ± 1.44 ^Ab^	11.61 ± 1.12 ^Bb^
0.1% fucoidan	3.95 ± 0.32 ^Da^	7.61 ± 0.63 ^Cc^	13.96 ± 1.06 ^Ac^	9.93 ± 0.92 ^Bc^
0.3% fucoidan	3.12 ± 0.27 ^Db^	6.09 ± 0.54 ^Cd^	11.21 ± 0.97 ^Ad^	8.40 ± 0.83 ^Bc^
0.5% fucoidan	2.36 ± 0.19 ^Cb^	5.82 ± 0.45 ^Bd^	9.10 ± 0.69 ^Ae^	6.33 ± 0.53 ^Bd^

*Note*: Dissimilar capital letters in each row disclose statistical difference about *p* < .05. Dissimilar small letters in each column disclose statistical difference about *p* < .05.

### 3.3 Oxidative stability

Table 3 shows the oxidative stability of butter samples of the control group and samples treated with different concentrations of fucoidan at 110 and 160 degrees Celsius. (*p* < .05). Based on the results, with the increase in the amount of fucoidan, the stability rate also increased (22.8 ± 0.013–37.8 ± 22.012 (h/min)) (*p* < .05). The stability of the product was dependent on the fucoidan dose and increased with increasing fucoidan concentration. Hydroxyl present in the reaction environment increases the probability of donating hydrogen to free radicals and subsequently increases the inhibitory power of the extract. By increasing the amount of fucoidan added to butter, its antioxidant property increases, so the highest antioxidant property was related to the sample with the highest amount of fucoidan. Another study also showed that with the addition of rosemary and chicory extracts, the amount of antioxidant properties was higher, making it more stable (Martínez‐Tomé et al., 2022), which was consistent with the results of this article. However, with the increase in temperature, the stability level decreased compared with the temperature of 110 degrees Celsius ((22.8 ± 0.013–37.8 ± 0.012). Taking advantage of the high antioxidant properties of fucoidan, it is recommended to consume the product fresh.

**TABLE 3:** Oxidative stability (Rancimat) of butter samples treated with diverse concentrations of fucoidan.

Butter treatments	110 (time(h/min))	160 (time(h/min))
Control	18 ± 0.03 ^D*a**^	10.8 ± 0.020
0.05	18 ± 0.010	10.8 ± 0.010
0.1	18 ± 0.010	10.8 ± 0.010
0.3	15.6 ± 0.020	9.8 ± 0.010
0.5	37.8 ± 0.012	22.8 ± 0.013

*Note*: Dissimilar capital letters in each row disclose statistical difference about *p* < .05. Dissimilar small letters in each column disclose statistical difference about *P* < 0.05.

### 3.4 Sensory properties

**FIGURE 1:**
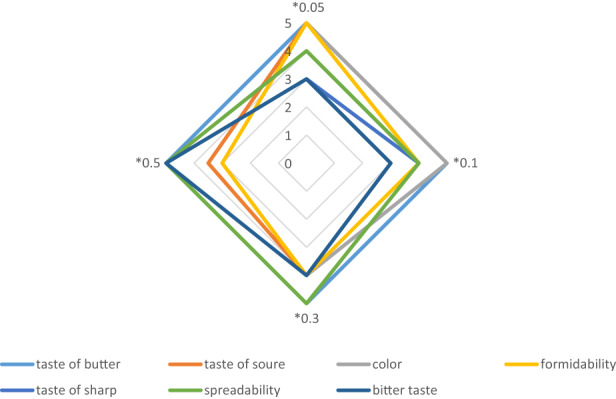
Sensory properties of butter samples treated with diverse concentrations of fucoidan.

Researchers have proposed simple methods such as peroxide index and acid number to determine the durability based on sensory tests (Huang, Hou, et al., 2022; Li, Wang, & Ye, 2022; Rahmati et al., 2022). Figure 1 shows the sensory evaluation of butter samples of the control group and samples treated with different concentrations of fucoidan on different days. The results showed that the butter samples with 0.5% fucoidan significantly reduced the amount of acid and peroxide and increased the shelf life of the butter based on the sensory test. No change in the taste of the butter was observed on day zero. Higher concentrations of fucoidan did not affect butter flavor. As the concentration of fucoidan increased, the color of the butter became slightly lighter. By increasing the concentration of fucoidan, the texture of the butter softened and spreadability increased. On the 40th day, the sharpness in the taste (rancidity) of the samples increased slightly, with the highest degree of sharpness detected in the control samples. Conversely, samples with the highest concentration of treatments showed no unpleasant organoleptic properties. The durability of food products can be considered as the period of time during which a product can be stored until it becomes unacceptable from a safety, nutritional or sensory point of view (Augustyńska‐Prejsnar et al., 2022; Pittia & Heer, 2022). Estimation of the shelf life of food and beverage products has become increasingly important in recent years due to technological advances and the increasing interest of consumers to eat fresh, safe, and high‐quality products. The shelf life of most food products is determined by changes in their sensory characteristics (Farooq et al., 2021). Therefore, in order to maximize the time to market while ensuring product quality, food companies must rely on accurate methods for sensory estimation of shelf life. The research showed that the food and beverage sections of a supermarket, where the number of food products, and their shelf life depend on their sensory properties, are far more than the products whose shelf life depends on microbiological or nutritional properties (Demarco et al., 2022). Usually, the oxidation state of the sample is checked along with sensory evaluation because the production of unpleasant taste and odor compounds is a secondary product of fat oxidation (such as pentanal, hexane, octanal) (Gyawali et al., 2022; Modesti et al., 2022). Therefore, fucoidan, especially at higher concentrations, can be used as an antioxidant agent for food preservation. Kordjazi et al. (2019) showed that the total phenolic content of fucoidan extracted from *S. angustifolium* is 9.73 ± 0.00 mgGAE/g which showed its strengthening antioxidant effects. The total phenolic and antioxidant content of *S. angustifolium* was reported as 0.061 ± 0.0001 mg/g and 0.231 ± 0.01 IC50 mg/ml, respectively (Kordjazi et al., 2019). Similar findings were reported by (Besednova et al., 2014) (Costa et al., 2010) (Im Lee & Seo, 2011), (Sannicolò et al., 2011), (Athukorala et al., 2006). High antioxidant effects of fucoidan extracted from seaweed have also been reported from Brazil (Fidelis et al., 2019), New Zealand (Koh et al., 2019), India (Jing, 2014), Malaysia (Lim et al., 2014), Nigeria (Ajisaka et al., 2016), and Korea (Kwon et al., 2011). The Sensory evaluation identifies flavor and odor defects in foods that are not usually detectable by instrumental tests (Owusu‐Apenten & Vieira, 2023). However, this requires trained personnel, adequate facilities, and high cost (Owusu‐Apenten & Vieira, 2023). In the results of the sensory test, there was no significant difference in the taste of the treated butter with the control butter on the first day, but in the following days, the control sample showed more sour taste, but the 0.5% treatment showed the least effect. On the 40th day, the pungent taste of the control butter slightly increased from the first day, but it had no effect on the final taste, so it was consistent with the increase of peroxide in the samples. The spreadability of the treatments increased compared with the control butter. Fucoidan is extracted from edible brown seaweed and does not have a bad effect on the taste of products (Remya et al., 2022). Fucoidan can be considered a functional food (Donn et al., 2022). Researchers' studies showed that by adding fucoid, product shelf life increased, and fucoidan has the most antioxidant effects and can be used as a food preservative (Chew et al., 2008; Kwon et al., 2011; Velasco et al., 2010). Various studies have focused on the presence of foodborne bacteria in samples of butter and dairy products made from contaminated milk (Ortiz et al., 2006; Velamakanni et al., 2022). Others have reported antimicrobial effects on fucoidan extracted from *S. angustifolium* (Benkirane et al., 2022; Buczek et al., 2022; Patel et al., 2022). Therefore, adding fucoidan isolated from *S. angustifolium* to butter due to its high antibacterial effects can reduce the risk of food pathogens and increase the shelf life and be a functional food.

## 4 CONCLUSION

In this study, the shelf life of fucoid‐treated butter was evaluated according to sensory evaluation and chemical properties of acid value and peroxide value and the relationship between these two parameters. Samples were kept for 60 days. In addition, fucoidan at higher concentrations increased the shelf life and sensory and oxidative stability of butter. Therefore, with decreasing acidity and peroxide, its shelf life and acceptance increased. The effects of fucoidan isolated from the seaweed *S. angustifolium* were dose‐dependent and due to the tastelessness of fucoidan, this amount could not change the taste of butter. This is a pilot study on the use of fucoidan isolated from the seaweed *S. angustifolium* as a food preservative to extend the shelf life of butter samples and to use it as a Functional food.

## CONFLICT OF INTEREST STATEMENT

Authors declared that they have no conflict of interest.

## Data Availability

The data that support the findings of this study are available on request from the corresponding author. The data are not publicly available due to privacy or ethical restrictions.
